# The design and development of a complex multifactorial falls assessment intervention for falls prevention: The Prevention of Falls Injury Trial (PreFIT)

**DOI:** 10.1186/s12877-017-0492-6

**Published:** 2017-06-01

**Authors:** Julie Bruce, Shvaita Ralhan, Ray Sheridan, Katharine Westacott, Emma Withers, Susanne Finnegan, John Davison, Finbarr C. Martin, Sarah E. Lamb, on behalf of the PreFIT Intervention (MFFP) Group, Sarah E. Lamb, Finbarr Martin, Ray Sheridan, Shvaita Ralhan, Katharine (Kitty) Westacott, John Davison, Ruma Dutta, Fiona Shaw, Jonathan Treml, PreFIT Study Group, Sarah E. Lamb, Martin Underwood, Finbarr Martin, Lucy Yardley, Dawn Skelton, Keith Willett, Sandra Eldridge, Tim Friede, Claire Hulme, Anne-Marie Slowther, Sarah Duggan

**Affiliations:** 1 Warwick Clinical Trials Unit, Division of Health Sciences, University of Warwick, Coventry, CV4 7AL UK; 2 Oxford University Hospitals NHS Trust, The John Radcliffe Hospital, Headley Way, Headington, Oxfordshire, OX3 9DU UK; 3 Royal Devon & Exeter Hospital, Barrack Road, Exeter, EX2 5DW UK; 4 University Hospitals of Coventry and Warwickshire, Clifford Bridge Road, Coventry, CV2 2DX UK; 5 Falls and Syncope Service, Newcastle upon Tyne Hospitals NHS Foundation Trust, Newcastle, NE1 4LP UK; 6 Guys and St Thomas’ NHS Foundation Trust, St Thomas’ Hospital, London, SE1 7EH UK; 7 Botnar Research Centre, Nuffield Department of Orthopaedics Rheumatology & Musculoskeletal Sciences, University of Oxford, Windmill Road, Oxford, OX3 7LD UK

**Keywords:** Falls, Falls prevention, Multifactorial assessment, Older adults, Trial intervention

## Abstract

**Background:**

This paper describes the design and development of a complex multifactorial falls prevention (MFFP) intervention for implementation and testing within the framework of a large UK-based falls prevention randomised controlled trial (RCT).

**Methods:**

A complex intervention was developed for inclusion within the Prevention of Falls Injury Trial (PreFIT), a multicentre pragmatic RCT. PreFIT aims to compare the clinical and cost-effectiveness of three alternative primary care falls prevention interventions (advice, exercise and MFFP), on outcomes of fractures and falls. Community-dwelling adults, aged 70 years and older, were recruited from primary care in the National Health Service (NHS), England.

**Results:**

Development of the PreFIT MFFP intervention was informed by the existing evidence base and clinical guidelines for the assessment and management of falls in older adults. After piloting and modification, the final MFFP intervention includes seven falls risk factors: a detailed falls history interview with consideration of ‘red flags’; assessment of balance and gait; vision; medication screen; cardiac screen; feet and footwear screen and home environment assessment. This complex intervention has been fully manualised with clear, documented assessment and treatment pathways for each risk factor. Each risk factor is assessed in every trial participant referred for MFFP. Referral for assessment is based upon a screening survey to identify those with a history of falling or balance problems. Intervention delivery can be adapted to the local setting.

**Conclusion:**

This complex falls prevention intervention is currently being tested within the framework of a large clinical trial. This paper adheres to TIDieR and CONSORT recommendations for the comprehensive and explicit reporting of trial interventions. Results from the PreFIT study will be published in due course. The effectiveness and cost-effectiveness of the PreFIT MFFP intervention, compared to advice and exercise, on the prevention of falls and fractures, will be reported at the conclusion of the trial.

**Electronic supplementary material:**

The online version of this article (doi:10.1186/s12877-017-0492-6) contains supplementary material, which is available to authorized users.

## Background

Falls and fall-related injuries are a major global public health burden, leading to loss of independence, disability and psychological distress. Although most falls result in minor injury, a third of people who fall sustain moderate to severe injury [1]. The World Health Organization rank fall-related injuries as the third leading cause of ‘years lived with disability’ [2]. Risk of falling and sustaining injury increases with age; data from the Centres for Disease Control and Prevention (CDC) show that injurious falls are a leading cause of death in those aged 75 years and over [3]. The cost of falls and fractures is substantial; direct health care and associated social care costs in the UK have been estimated at £2 billion per annum, mostly associated with hip fracture [4]. In the US, the annual direct and indirect medical costs of caring for fall injuries is projected to rise to $47 billion dollars by the year 2020 [5].

Falls have a multifactorial aetiology and numerous risk factors have been identified [6] The risk factors are diverse, including impairments of gait and balance, visual impairments, syncope and cardiac arrhythmias, polypharmacy, foot disorders and environmental hazards. Considerable effort has focused on the evaluation of fall prevention strategies: early trials of assessment and treatment of multiple risk factors, termed multifactorial falls prevention (MFFP) strategies, were initially promising, suggesting up to a 30% falls reduction when compared to usual care [7]. These early trials provided the foundation for the mandatory establishment of secondary prevention in the UK, through the introduction of falls services to undertake MFFP on those with a history of falling [8]. To date, however, there is limited evidence of efficacy of MFFP on outcomes of injurious falls, such as fracture [9]. To justify the introduction of widespread services for the primary prevention of falls, evidence is needed on whether the preventive approach works, also whether multifactorial interventions reduce injurious falls.

### The prevention of fall injury trial (PreFIT)

**Fig. 1:**
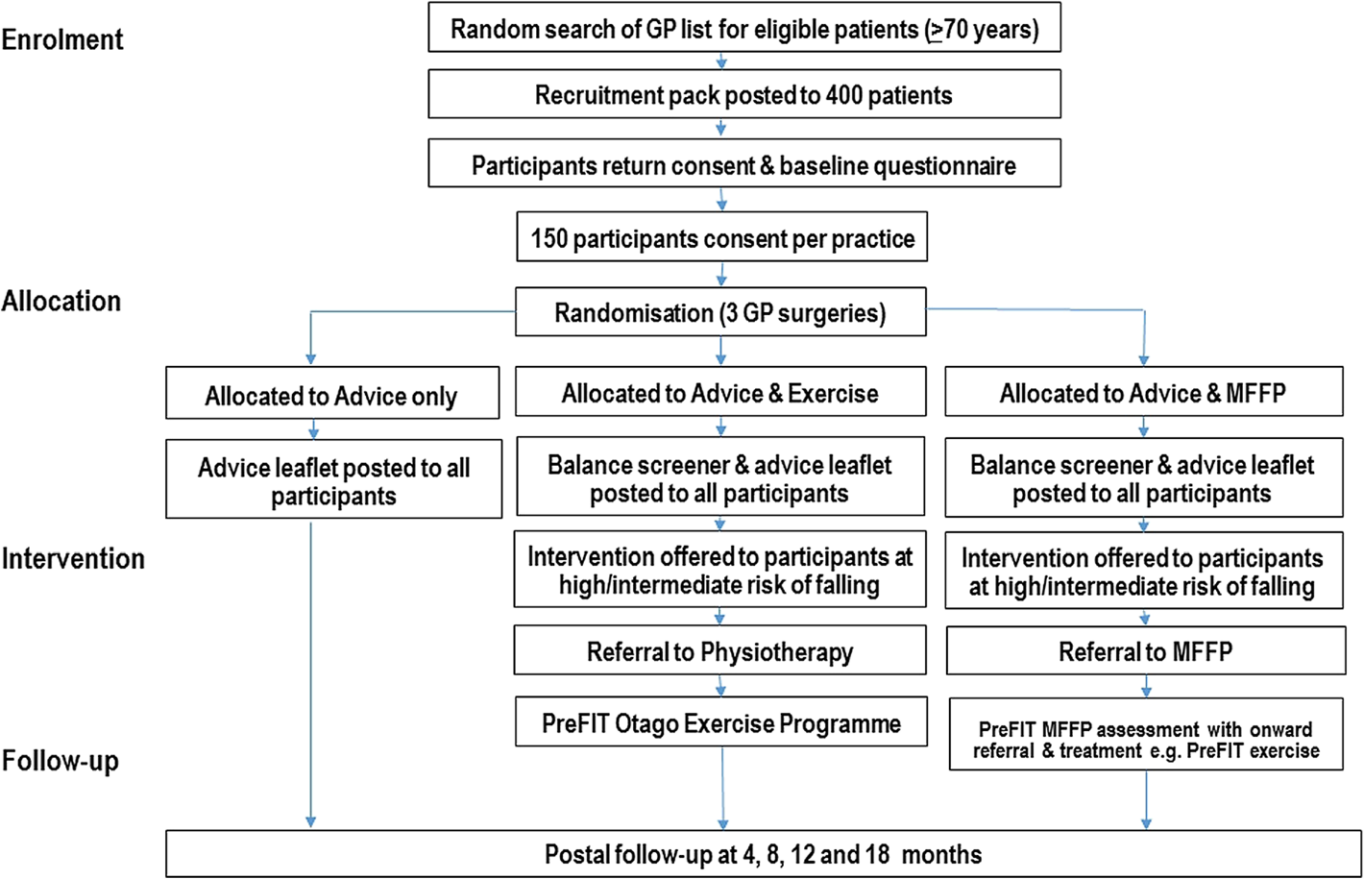
Flow diagram of cluster trial design

In 2010, the National Institute of Health Research (NIHR) Health Technology Assessment (HTA) programme commissioned a large multicentre pragmatic cluster randomised controlled trial (RCT) to evaluate the clinical and cost-effectiveness of three alternative primary care interventions for preventing falls and fractures (ISCTRN 71002650). The Prevention of Fall Injury Trial (PreFIT) compares three interventions of advice alone, versus advice supplemented with either exercise or MFFP, in community-dwelling adults aged 70 years or older. The unit of randomisation is the General Practice. The rationale and methodology for PreFIT is fully described within a detailed published protocol [10]. In brief, the advice only intervention is the ‘Staying Steady information leaflet published by AgeUK [11] and the exercise intervention is based upon the Otago Home Exercise Programme which targets lower leg strength, balance and walking. The Otago programme has been evaluated in different settings and populations [12]. The MFFP intervention is the focus of this manuscript. Participant referral to ‘active’ treatments of exercise or MFFP is based on a primary care based screening approach, determined from a short self-completed falls and balance screening survey. General practices randomised to deliver exercise or MFFP send out the postal screener and refer responding participants to treatment based upon their history of falls and balance problems. The trial outcomes are fractures, falls, quality of life and healthcare resource use. Figure 1 displays the Consort flow diagram and overall study design for the trial.

Our aim was to develop a high quality evidence-based intervention suitable for delivery and testing within a pragmatic trial conducted in the NHS primary care setting. As per Medical Research Council (MRC) guidance for the development and evaluation of complex intervention trials and calls for improved reporting of trial interventions [13], we describe the development and procedures for the MFFP intervention, adhering to the recommended Template for Intervention Description and Replication (TIDieR) guidance [14] [Additional file 1].

## Methods

### Overview of the PreFIT MFFP intervention

A multifactorial intervention is defined as that where ‘each individual receives an assessment of known risk factors for falling and receives an intervention matched to their risk factor profile’ [15]. Selection of risk factors for inclusion within the PreFIT intervention was informed from the existing evidence-base at the time of development, including systematic reviews and clinical guidelines e.g. American Geriatrics Society (AGS), British Geriatrics Society (BGS) and the UK National Institute for Health and Care Excellence (NICE) [1, 8, 16]. We elicited a range of expert views, including from the British Geriatrics Society and practice experts within the field of falls and bone health. An MFFP intervention must be feasible to deliver within busy NHS clinical environments, thus, as well as content, consideration was also given to practical challenges of staffing, time, space restrictions, expertise, equipment and resources. A member of the trial team (JB) observed falls history interviews and clinical assessments conducted by experienced consultant geriatricians on hospitalised fallers. The findings were used to inform the trial intervention. The final intervention was clearly documented and fully ‘manualised’ for reference purposes, both to promote consistency in delivery across multiple trial sites and to enable future replication. After development, the draft intervention was delivered to participants recruited from one general practice in the pilot region (Devon). Following minor revisions, the MFFP intervention was then implemented within the main trial and administered by other participating sites across England.

### Evidence for MFFP

We considered evidence of effectiveness and settings in which trials had been conducted. Although update reviews have since been published, at the time of intervention development, 34 RCTs of multifactorial intervention were included in a large Cochrane systematic review investigating strategies for preventing falls in elderly people [17]. A separate systematic review and meta-analysis examined the effectiveness of multifactorial assessment intervention programmes where participants were recruited from primary care, community and emergency department (secondary prevention) settings [9]. Of the 19 trials included in the review by Gates et al. [9], 13 trials recruited from the primary care or community setting. Of these, seven targeted high-risk populations (e.g. fall in last 3 months) and six trials recruited a broad unselected population of older adults. Although several trials captured outcomes of injurious falls, only one clearly reported fracture outcomes [9, 18]. At the time of development of PreFIT, only four trials of MFFP had been conducted in the UK NHS setting, all recruiting fallers from emergency departments – no UK trial had recruited all participants from primary care.

An American clinical guideline and systematic review with a narrower focus on outpatient approaches to falls prevention classified MFFP as ‘comprehensive’ or ‘non-comprehensive’. Comprehensive management was considered active management of fall risk factors and conditions identified in the assessment, whereas non-comprehensive, related to partial or limited management of identified fall-related risk factors [19]. However, the authors acknowledged that characteristics of comprehensive multifactorial assessment have not been clearly defined.

These systematic reviews reveal that completed trials testing multifactorial interventions have varied in terms of location of case finding approach (and therefore probably casemix of participants), delivery, skill mix, assessments conducted and interventions offered. Clinical and methodological heterogeneity hampers the interpretation of pooled statistical analyses, although evidence from the multiple systematic reviews suggests that multifactorial interventions reduce the rate of falls (number of events) but not the risk of falling (number of fallers). One challenge is the complexity of these multifactorial interventions; when a trial demonstrates a positive effect or clinically important reduction in falls, the very nature of a heterogenous falls intervention prevents the elucidation of the exact successful components. Published research often fails to describe individual components sufficiently, making it both difficult to elucidate the main driver of effect but also to replicate potentially successful components in clinical practice. The systematic review of MFFP trials reported by Gates et al. [9] summarised interventions as e.g. ‘geriatric assessment, vision, drug check’ as description of content within many of the trials was limited. There is no clear evidence from systematic reviews which core risk factors within the ‘black box’ of MFFP are likely to drive any effect*.*

Multifactorial risk assessment, followed by targeted treatment of individual risk factors, is currently the mainstay for falls prevention in the UK. Higher intensity interventions that provide treatments to address identified risk factors (such as supervised exercise for balance problems), are thought to be more effective than information and referral alone [9]. Guidance from the AGS, BGS and NICE recommend that older adults at high risk of falls receive an MFFP assessment and individualised, targeted interventions to address risks and deficiencies identified in the assessment. The key risk factors are described in the next section.

### Participant screening and referral to MFFP

The decision to refer a participant for a PreFIT MFFP assessment is determined from a 1-page short self-complete balance and falls screening survey designed to identify people at higher risk of falling. This screener, mailed from and returned to general practices, includes questions on falls history in the last 12 months and current balance problems whilst walking, dressing, toileting or taking a bath. Items were selected based on previous research predicting thresholds for future falls risk [20]. Participants with a history of falls or current balance problems are offered the opportunity to attend for further assessment and treatment.

### Overview of MFFP assessment

**Fig. 2:**
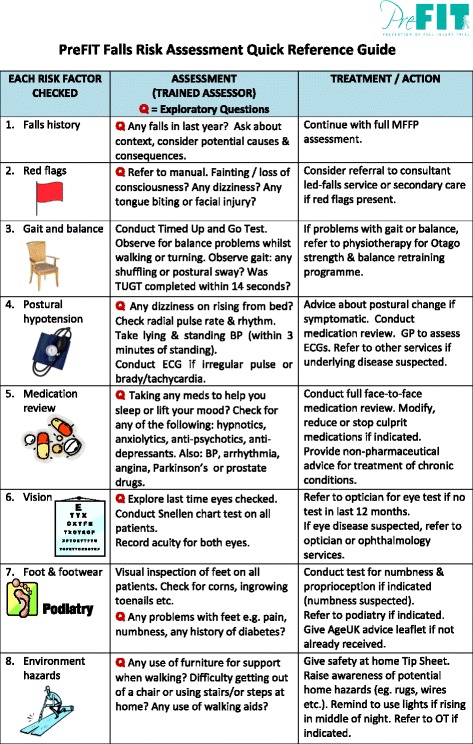
PreFIT Falls Risk Assessment Quick Reference Guide

The PreFIT falls assessment consists of examinations performed within the general practice, the home, community or general hospital by a practice nurse or equivalent registered healthcare professional, or by a community or hospital-based falls team (Table 1). The location and person responsible for the falls assessment will vary due to the pragmatic trial design and availability of local services. Appointments with participants are booked for 1 h. The trained assessor undertakes a detailed falls history interview with consideration of ‘red flags’; followed by assessment of balance and gait; vision; medication screen; cardiac screen; feet and footwear screen and home environment assessment. Every risk factor is assessed in every trial participant referred for MFFP. Assessment is followed by recommendations or further onward referral to another service where indicated. The assessor completes trial documentation and arranges onward referrals e.g. to consultant-led falls service, physiotherapy, GP-led medication review, occupational therapy etc., as per our recommended treatment pathways for each risk factor. Assessment procedures and brief justification for the selection of each of the risk factors is described in more detail below (Table 2). A quick reference pictorial guide is presented in Fig. 2.

**Table 1:** Overview of PreFIT MFFP intervention, as per TiDIER [12] criteria

TiDIER criteria ^(12)^	Description of PreFIT falls assessment and quality control procedures
*Staff training and participant referral*	
Who provided training	Consultant Geriatrician or Specialist Registrar in Geriatrics/Elderly Medicine with expertise in falls assessment delivered 5 h MFFP training.
Who received training	Primary care practice nurses and consultant-led falls team comprising trained healthcare professionals (e.g. registered nurse, occupational therapist or physiotherapist).
Participants receiving MFFP intervention	Trial participants aged 70 years or older, randomised to MFFP arm. Decision regarding eligibility for MFFP assessment based upon history of falls and balance problems.
Referral procedure	Participant invited to attend for 1-h individual ‘health assessment’ by general practice or falls team or service, depending upon locality. Written letter to confirm appointment location, time and date if this was local practice.
*Assessment Procedure*	
Materials required	Metal tape measure*, stopwatch*, hard-backed arm chair of 40-50 cm height, Snellen chart (3 m)*, eye patch, calibrated manual or electronic sphygmomanometer, ECG machine, cotton wool balls for podiatry assessment.
Where	Falls assessment undertaken in suitable location with a quiet room. Access, parking and transport should be considered. A pragmatic approach was taken to select a location appropriate for each region or cluster e.g. general practice, community hospital or falls service, depending upon availability. The room must be of a comfortable temperature with ‘do not disturb’ signage on the door. Room must have bed or plinth with footstool to allow patient to lie in supine position. Correct distance for the TUGT and Snellen chart vision assessment clearly marked using floor tape.
When	Single 1-h assessment at time suitable for participant and assessor.
Tailoring	Every risk factor assessed on every participant. Additional assessment and referral arranged in the event of risk factor identified or suspected (see Table 2). Referral pathways can be tailored to local setting e.g. referral to NHS chiropody/podiatry if service available. Location of assessment and staff background varied between and within participating regions.
Modifications	Modifications were made to data collection forms during the pilot study. Minor adaptations included production of additional laminated materials as visual aids e.g. listing of psychotropic and culprit medications to aid drug screening.
*Intervention Fidelity*	
How well – Training	Training Evaluation Forms completed by staff trained in MFFP intervention - asked to return anonymously using stamped addressed envelope to Trial Office. Asked to report on quality of MFFP training (presentations, content, risk factor assessment procedures, documentation, safety reporting, roles & responsibilities). Provided with free-text sections to comment on: whether to spend more or less time on particular aspects; confidence in delivering individual components of the intervention; quality of Therapist Reference Manual; data collection forms and overall rating of training delivered (very poor, poor, average, good or very good).
How well – Intervention delivery (Who)	Training emphasises adherence to the PreFIT standardised protocol. Quality Control (QC) visits to staff at every site undertaken by member of PreFIT team, Consultant Geriatrician or Specialist Registrar in Elderly Medicine. QC visit includes observation of trainee conducting 1-h MFFP assessment, with consent of participant. Aim to observe at least one MFFP assessment per trainee.
How well – Intervention delivery (What)	5-page QC Assessment Form completed covering: accuracy of completion of trial paperwork; 15-point checklist of risk factors; whether any further referrals were warranted and actioned appropriately; whether the MFFP assessment was satisfactory or unsatisfactory (follow-up visit required). Also whether any serious concerns were identified that required reporting to Intervention Lead and/or any areas requiring further training. QC form signed and dated by assessor and trainee.

*Provided by PreFIT team

**Table 2:** Overview of PreFIT MFFP risk assessment and recommended treatment referral pathways

Component	Screening questions and overview of procedure	Referral pathway
Falls History	Introduce yourself and explain purpose of the appointment. Use exploratory screening questions to initiate discussion. Explore balance difficulties with non-fallers. Conduct full history with fallers using questions from Table 3. (Therapist Manual provides more detailed advice e.g. use clear language and explanations, develop skills to follow relevant leads, incorporate open exploratory questions and allow the older person to tell their ‘story’ without rushing or interrupting them.) Explore specific falls and also near-miss events. *Q. Have you fallen the last 12 months?* *Q. Do you have any difficulties with your balance whilst walking or dressing?*	Refer to Falls Service Doctor (Consultant Geriatrician), GP or other speciality depending upon risk factor identified. Notify GP of any red flags identified during assessment. Record date, service and name of person referred to.
Red Flags	A “red flag” is a warning sign of more serious underlying medical causes. Red flags indicate that referral to a GP or medical specialist is warranted e.g. bradycardia, history of near fainting or syncope. Any symptoms suggestive of seizure activity e.g. visual aura, tongue biting. There is no single question or validated algorithm for taking a comprehensive falls history, it requires good listening skills and ability to link different risk factors to each other. Ask ALL questions in Table 3 of those who have fallen previously.	
Balance and Gait	Conduct Timed Up and Go Test (TUGT) [22]. Observe gait whilst walking and turning. Observe for signs of unsteadiness, shuffling walk, uneven stride length, veering or grabbing for furniture. Any TUGT ≥14 s, gait problems *or* fear of falling requires referral to PreFIT physiotherapist.	Referral to PreFIT physiotherapist to initiate PreFIT exercise programme.
Postural hypotension	*Q. Do you ever feel dizzy or lightheaded if you stand up too quickly?* *Q. Do you ever feel dizzy or lightheaded first thing in the morning when you get out of bed?* Screen for postural hypotension. Regardless of response to screening questions, check heart rate and rhythm, conduct lying and standing blood pressure (BP). Use recently calibrated manual or electronic sphygmomanometer. Explain procedure; ask participant to lie on couch. Wait 2-3 min before taking first BP reading. Record radial pulse and assess rate/rhythm: sinus bradycardia (<50 bpm), sinus tachycardia (>100 bpm). Take lying BP and record. Ask to stand, repeat measurement on same arm, as soon as standing and again within 3 min of standing. Record measurement. Patient has symptoms and *any* of the following between 1 to 3 min of standing up:- Test positive if drop in systolic BP of at least 20mmHG; Test positive if drop in systolic BP <100 mmHg; Test positive if drop in diastolic BP of at least 10mmHG. *ECG:* An electrocardiogram (ECG) should be undertaken on anyone with an irregular pulse, bradycardia or tachycardia. If possible, use an electronic ECG machine with a printed report.	If symptomatic postural hypotension: - Conduct full medication review and consider culprit drugs e.g. anti-hypertensives, vasodilators, CNS drugs etc. - Change timing of diuretics to avoid nocturnal micturition. - Give PH information leaflet Consider referral to consultant-led falls service if arrhythmia with syncope. ECG should be interpreted by the GP, doctor, specialist nurse or trained cardiac technician. ECG findings inform decision about treatment or referral for further assessment e.g. cardiology or medical referral.
Medication review	*Q. Are you taking any medications to help you sleep?* *Q. Are you taking any medications to lift your mood?* A visual review of all prescribed drugs combined with face to face discussion conducted on all patients (Level 1). Any patient prescribed one or more of the following drugs referred for Level 3 comprehensive GP-led medication review:- . *Psychotropic and related drugs:* antidepressants, psychotropics, sedatives, and anti-manic. Hypnotics and Anxiolytics (Night Sedation – British National Formulary Section 4.1), Antipsychotics (Section 4.2), Antidepressants (Section 4.3). *Culprit drugs* Cardiovascular (Section 2), Diuretics (Section 2.2), Anti-arrhythmia (Section 2.3) Beta-adrenoceptor blocking (Section 2.4), Hypertension and heart failure (Section 2.5), Nitrates, calcium-channel blockers & others (Section 2.6), Drugs used in Parkinsonism & related disorders (Section 4.9).	GP to conduct medication review if prescribed any psychotropic or culprit medication.
Vision	*Q. Have you had your eyes checked by an optician in the last 12 months?* *Q. Has your eyesight changed or have you had any problems with your vision since your last appointment with the optician?* Other exploratory questions include:- *Q. any problems with reading?* (suggests problem with near vision) *Q. Any problems with watching TV?* (suggests problem with distance vision) *Q. Do you wear bifocal glasses?* The Snellen Chart should be wall mounted and in a well-light position. The person should stand EXACTLY 3 m from the chart (adjusted for 6 m), distance calculated and marked with tape on the ground. Can wear distance vision glasses, cover one eye with patch and ask to read down chart until they reach the smallest line of letters they can distinguish on the chart. Conduct on both eyes. Any visual acuity at less than 6/6 requires referral to optician for eye test. Other advice includes wearing of bifocals/multifocals whilst walking outdoors should be avoided; taking care when wearing new spectacles [28].	Encourage all participants to attend free eye check. If had eye test in last 12 months but vision has deteriorated, ask to make optician appointment. If eye disease or cataracts suspected, refer to optician. If visual impairment, consider home environment assessment and referral to occupational therapy.
Foot problems	*Q. Do you have any problems with your feet?* *Q. Any pain in your feet?* *Q. Any numbness in your feet?* *Q. Do you have diabetes?* *Q. Do you attend chiropody / podiatry services?* Visual examination of feet to check for bunions, hammertoes, calluses or in/overgrowing nails that may cause pain or gait disturbances [32]. Conduct proprioception check if concerned about numbness or food positioning (refer to manual). Assess footwear and give advice on recommended shoes (supportive heel collar, heel height of less than 2 cm, slightly bevelled heel, fastened using laces, straps or buckles, thin firm midsole to allow sensory input, slip resistant sole and wide fitting [33].	Refer to local podiatry or chiropody services if available. Consider referral to physiotherapy for balance retraining if concerned about gait style or foot placement. Give AgeUK advice leaflet. Consider referral to secondary care services if indicated e.g. diabetic services.

Mandatory questions are italicized

### Content of PreFIT MFFP assessment

#### Falls history interview/red flags

A comprehensive falls history interview is undertaken with every participant invited for assessment. The purpose of a falls history interview is to identify any predisposing factors leading to a fall, to explore context and consequences of any previous falls or near misses to provide clues about causation. The PreFIT assessor is trained in systematic enquiry about falls, including symptoms and contextual factors before, during and after any fall-related ‘event’ including trips and stumbles. Taking a good falls history is an important skill which can be honed over time although there is no single question or validated algorithm to follow. Clinical guidelines do recommend that detailed falls history interviews should be undertaken [16, 21], however we found no templates or guidance for the types of questions to include. We therefore generated a template list of PreFIT prompt questions based upon observations of experienced clinical staff conducting during falls interviews with hospitalised older adults. The prompt questions were produced as an easy-to-use laminate for PreFIT assessors (Table 3). The aim of a falls history interview is to identify intrinsic risk factors, relevant activities and environmental challenges, any of which may be amenable to modification. Participants may have a combination or mosaic of risk characteristics, which requires careful exploration and the ability to *link* different risk factors e.g. visual problems leading to repeated trips and stumbles within the home. “Red flags” are warning signs that referral to a GP or medical specialist is warranted e.g. cardiac abnormalities, history of syncope, evidence of seizure activity such as aura or tongue biting etc.

**Table 3:** Questions to ask during PreFIT MFFP assessment falls interview

Question	Possible/probable cause of falls & onward treatment pathway
Any dizziness or giddiness?	Dizziness or giddiness defined as feeling dizzy or light-headed, as if going to faint. Ask about circumstances. Check for postural hypotension (refer to manual).
Any vertigo?	A sensation of spinning. May represent vestibular disease which requires medical diagnosis.
Any muscle weakness in the legs? Is one leg weaker than the other?	If the person has one leg weaker than the other, this requires a full medical review. Refer to consultant-led falls service or secondary care.
Any sudden loss of consciousness?	Any sudden, unexplained loss of consciousness (syncope) requires a medical review. Reasons may include anything from a vasovagal faint to a cardiac arrhythmia or other cardiac problem. Requires referral to secondary care consultant-led falls service.
Any palpitations or angina?	Refer to definitions. Suggestive of cardiac disease. Ask about exercise-related chest pain. The first stage for referral is to the GP unless the pain is present at time of assessment (if so, urgent referral to secondary care for cardiac assessment.).
A trip or stumble on a hazard? Explore circumstances.	Ask about home environment. Use home environment screening questions.
Any rapid position change?	May indicate postural hypotension or if head movement, may indicate carotid sinus hypersensitivity. Continue with falls assessment and consider referral to consultant-led falls service/ secondary care. This may also indicate visual dependency for stability due to vestibular insufficiency (with or without vertigo).
Any visual disturbance, such as blurred vision?	May indicate epileptic fit or may indicate visual problems associated with tripping on hazard. Continue with assessment also conduct vision check.
Any injuries sustained from the fall, bruising, fractures etc.?	May indicate sudden drop and unable to protect themselves. Continue with falls assessment and consider other circumstances.
Any facial injuries?	Similarly, indicative of sudden fall and unable to protect themselves. Continue with falls assessment and consider referral to consultant-led falls service/ secondary care.
Any tongue biting?	Suggestive of epileptic fit. Ask about incontinence. Refer in the first instance to the GP who may refer to consultant-led falls service/secondary care.
Were they wearing a very tight collar around the time of the fall?	Indicative of carotid sinus hypersensitivity. This will require referral to a consultant-led falls service.
Have they ever been incontinent when/after falling?	May indicate epileptic-type seizure. Enquire about tongue biting. Consider referral to consultant-led falls service.
Do you worry about your balance?	May indicate fear of falling. May benefit from balance retraining and reassurance. Refer to PreFIT physiotherapist.

#### Balance and gait

The Timed Up-and-Go (TUGT) was developed as a basic test for functional mobility [22]. It consists of observation and measurement of the time taken for participants to stand up from a standard chair with arms of seat height 40-50 cm, walk forwards a distance of three metres at a normal walking pace, turn and walk back to sit down to the original sitting position. Time taken is recorded in seconds. Participants can push off using the chair arms if they need to [23]. Shoes are worn and the person’s usual walking aid can be used during the test. The exact distance is measured and marked on the floor using tape. Assessors are trained to observe for any gait-related problems on entry to the assessment room and during the TUGT. Observations are made whilst the participant is standing and walking e.g. noting stride length, foot clearance, veering to one side, grabbing or lunging for room furniture. A 14-s cut-point on the TUGT is predictive of falls in community-dwelling, frail older adults [23], therefore this threshold is used to generate onward referral to PreFIT physiotherapy for strength and balance retraining (the Otago Exercise Programme). For the purpose of the trial, we defined deficits as taking longer than 14 s on the TUGT, evidence of gait or balance problems or fear of falling (identified during the falls history interview).

#### Postural (orthostatic) hypotension

A standard definition for postural hypotension, agreed by a multidisciplinary consensus conference [24] is used: *“postural or orthostatic hypotension, defined as a sustained reduction of systolic blood pressure of at least 20 mmHg or a drop in systolic blood pressure to below 100 mmHg, or a reduction of diastolic blood pressure of 10 mmHg within 3 min of standing*”. PreFIT participants are screened by asking if they ever feel dizzy or light-headed if they stand up too quickly or first thing in the morning getting out of bed (Table 2). Lying and standing blood pressure is taken on all trial participants, regardless of history of dizziness, using a calibrated manual or electronic sphygmomanometer. The radial pulse is taken for 1 min and electrocardiogram (ECG) taken if the participant has an irregular pulse, bradycardia or tachycardia. Participants are asked to report the presence of dizziness or light-headedness during the standing phase. Symptomatic patients only are given a PreFIT postural hypotension leaflet that provides advice about changing position, fluid intake, tightening calves when getting up from a lying position etc. Onward referral is made to the GP or consultant-led falls clinic if any cardiac problem is suspected (Table 2).

#### Medication review

Polypharmacy is very common in older adults [7]. The UK Department of Health defines a medication review as “a structured, critical examination of a patient’s medicines with the objective of reaching an agreement with the patient about treatment, optimising the impact of medicines, minimising the number of medication related problems and reducing waste” [25]. This policy document described different levels of medication review (Level 0 to Level 3), relating to intensity of review, skill of assessor and whether or not conducted in the presence of the patient.

High-risk or ‘culprit’ medications that may contribute to risk of falls include drugs targeting the central nervous system, such as the psychotropics, benzodiazepines, antidepressants antipsychotics [26]. Other drugs, although the evidence base for the causal relationship with falls is slightly weaker, include antiepileptics, antiarrhythmics, urinary anti-cholinergics and alpha-blockers [26, 27]. For PreFIT, two types of medication reviews are conducted; firstly, a visual medication screen of all prescribed drugs combined with a face-to-face discussion with the patient about the use of prescribed and over the counter drugs (DoH Level 1). This initial screen searches for specific classes of high-risk medications based on a PreFIT listing of (a) psychotropics and (b) culprit medications (Table 2). Data are also recorded on prescribed bisphosphonate drugs. Psychotropic medications include any antidepressant, psychotropic, sedative/anxiolytic or antimania drugs. Any “culprit” medication includes antihypertensives, antiarrhythmics, diuretics, vestibular suppressants, analgesics, anticonvulsants, anti-Parkinsonians or vasodilators. Any patient prescribed one or more of these drugs is then referred for a Level 3 comprehensive GP-led clinical medication review [25]. This involves a separate appointment between the patient and GP, either face to face or by telephone if any medication revision is considered minor. One or more nominated GPs from each practice are given brief training on high-risk medications and how to conduct a falls-related medication review.

#### Visual acuity

Impaired visual acuity (sharpness or fine detail of vision), has been identified as a risk factor for falls in some, but not all, studies of older adults. The UK NICE concluded that there was insufficient evidence that single interventions targeting vision impairment alone were effective, but that referral for visual correction within a multifactorial intervention could contribute to falls reduction [8]. Bifocal glasses can add to the risk of falls because near-vision lenses impair distance vision and affect depth perception, affecting the ability of an older person to detect environmental hazards [28]. The wearing of multifocal/bifocal glasses should be restricted in older adults prone to falls. Given this evidence, we included a simple visual acuity screener using a standard 3-m Snellen chart test [29]. For PreFIT, the Snellen chart screening tool is used in conjunction with questions about last eye check, any changes in eyesight or any visual problems. Participants are asked to bring their spectacles to the appointment - distance vision glasses can be worn during the sight test. The person can stand or sit three metres from the chart; the distance is clearly marked on the floor using tape. Any visual screening test scoring less than 6/6 on either eye requires referral to an optician for a full eye test. Eye tests are free in the UK for anyone aged 70 years and older. PreFIT participants are therefore encouraged to attend their one free eye check per annum.

#### Foot problems

Up to a third of older people suffer from foot problems, such as foot pain, toe deformity, weakness or restricted range of motion [30, 31]. These problems are common reasons for attending primary care services. Other UK surveys suggest that the main foot conditions affecting older people requiring core podiatry include nail problems, corns, calluses and toe deformities [32]. PreFIT participants are screened for any foot problems including pain, numbness, diabetes and regular attendance at chiropody/podiatry. A visual examination is made to check for bunions, hammertoes, calluses or nails that may cause pain or gait disturbances. Tests are undertaken for proprioception (big toe positioning with eyes closed) and sensation, by brushing a cotton wool ball lightly across both feet (with the sternum used as normative reference). Assessment of footwear is undertaken and advice is given on proper fitting shoes e.g. wide fitting, low heel height, slightly bevelled heel, good supportive heel-collar, a thin firm midsole to allow sensory input and slip resistant sole [33].

#### Home environment

UK NICE guidance reports on different studies of domestic hazards, home hazard modification and safety interventions but concluded that in older adults without a history of falls in the previous year, there was no evidence of effectiveness [1, 8]. There is one good quality trial demonstrating that home hazard assessment with a supervised modification programme is effective in reducing falls in those discharged from hospital [34, 17]. The evidence suggests that home hazard removal and advice about functional activities is most effective in reducing falls in individuals with visual impairment [9]. It is not a prerequisite that a home assessment is conducted for *every* PreFIT participant, but if there are concerns about the home environment or safety whilst performing activities, or if suspicions are raised during the falls interview e.g. someone reporting repeated trips or stumbles at home. Onward referral is then made to occupational therapy or social services. In some instances, UK social services can cover the cost of home adaptations or equipment. Screening questions include asking about the use of furniture whilst walking, difficulty getting out of a chair or rising from the toilet, any stairs or steps at home, coping with stairs, use of walking aids. A PreFIT Home Safety Tip Sheet is given to anyone who may benefit from simple advice on home safety.

### Exclusions from assessment

We did not include detailed tests of urinary incontinence, hearing or osteoporosis risk, nor a comprehensive assessment of the neurological or cardiac systems (Table 4). The PreFIT falls assessment does include questions to explore urinary incontinence in relation to any fall or near miss event, (‘*whether any incontinence occurred before, during or after a fall’*). Assessment of hearing is not undertaken although the baseline participant questionnaire includes items on self-reported difficulty with hearing. At the time of developing the intervention, guidelines on the prevention and treatment of osteoporosis and the use of Vitamin D for fracture prevention were under review by NICE [8]. To our knowledge, none of the completed clinical trials of MFFP included screening of osteoporotic risk, although description of intervention content was inadequate [9]. We were cognisant of the different clinical backgrounds of assessors and of barriers to accessing trained medical practitioners in some settings. Safety was, therefore a consideration and we did not ask staff to conduct carotid artery stimulation to check for carotid sinus hypersensitivity.

**Table 4:** Components included/excluded from the PreFIT MFFP assessment

Included	Excluded	Rationale for exclusion
Assessment of:- Falls history Red flags Balance and Gait Postural hypotension Medications Vision Foot problems Environmental hazards	Hearing	Not recommended within NICE/AGS/BGS guidance (8,14). Screening questions about hearing difficulties included in baseline participant questionnaire.
	Osteoporosis	Risk assessment was not undertaken to avoid confounding between bone health and falls prevention interventions. NICE guidelines on prevention and treatment of osteoporosis and Vitamin D for fracture prevention were under revision at the time of intervention development. Prescription data on bisphosphonate medications and mineral supplementation were also collected from all participating general practices.
	Cognitive impairment	Patients with known severe cognitive impairment were excluded from study entry. No evidence that cognitive or behavioural interventions alone reduce the incidence of falls in community-dwelling older people [8].
	Neurological function	AGS/BGS guidance recommends assessment of neurological function, including cognitive evaluation, lower extremity peripheral nerves, proprioception, reflexes and tests of cortical, extrapyramidal and cerebellar function in older people. The PreFIT assessment includes a test of proprioception (toe movement) and a further test for numbness and sensation if foot numbness is suspected. It was not feasible to conduct more intricate tests of cerebellar function in the primary care setting.
	Carotid sinus hypersensitivity	Cardiac pacing is effective in reducing falls and syncope in adult fallers with cardio-inhibitory carotid sinus hypersensitivity. PreFIT assessment includes a check of heart rate, rhythm and postural hypotension. For safety reasons, we did not recommend that carotid artery stimulation be conducted in the community setting, where there was the potential for limited access to immediate clinical support.
	Urinary incontinence screening tool	The PreFIT falls intervention interview includes a list of question prompts, including enquiring about any incontinence occurring before, during or after a fall event.

## Results

### Delivery of the PreFIT MFFP assessment

#### Staff training

A detailed PreFIT Intervention Manual was given to every member of staff responsible for MFFP assessment. This comprehensive 115-page manual describes the scientific rationale and evidence-base for each risk factor, trial process and procedures, with flowcharts for treatment pathways for onward referral. Laminated easy-to-use instruction sheets were produced e.g. how to conduct the TUGT, vision check etc. Figure 2 is an example of a quick reference guide to the PreFIT falls assessment. Healthcare staff participating in the MFFP intervention received a 5-h structured training session in how to conduct the PreFIT falls assessment. Training was delivered during regular working hours, with time approved by relevant employers. Staff training could take place at any suitable location, such as a GP surgery, community venue or hospital setting, depending upon availability. Staff ranged from experienced falls team personnel (medical doctors, nurses, occupational therapists or physiotherapists) to general practice staff e.g. advanced nurse practitioners/practice nurses. Staff were required to have either a nursing or an allied healthcare background with professional registration. A medical geriatrician (RS/KW/SR) and senior researcher from the trial team provided training (JB). Additional training on ‘how to conduct a falls-related medication review’ was undertaken with one or more nominated GPs from each participating general practice. Modest reimbursement was given to practices willing to undertake PreFIT MFFP assessments, as per excess treatment costs agreed and approved by the funder.

#### Pilot study

A pilot study was undertaken to determine acceptability of the MFFP intervention to participants and clinicians, and to assess the feasibility of delivering the intervention in the NHS setting. A number of clinical teams and primary care staff from different regions had expressed interest in the trial, however staff from the Devon Region had the capacity to support the pilot study and support training of primary care staff. Twelve general practices in Devon were identified with support from the Comprehensive Local Research Network (now Clinical Research Networks) and regulatory approvals were obtained. Participant recruitment ran from July 2010 to March 2012. A total of 1801 participants were recruited from the 12 practices, of which four practices were randomised to deliver the MFFP intervention. Completed balance screeners were received from 492/575 (86%) participants within these four practices. Of responding participants, 190 were eligible for MFFP falls assessment. Findings from the balance screener revealed that *n* = 55 (29%) had fallen more than once in the previous year, *n* = 111 (58%) reported a single fall with or without balance problems, and *n* = 24 (13%) reported balance problems only. Of the 190 participants invited for assessment, 148 attended (78%). The draft MFFP intervention was administered to participants within one general practice; after minor revisions and improvements to data collection forms, the modified intervention was then rolled out to the remaining three practices. After external independent review of pilot study data and approval from the funder, the main trial was launched. Full data on assessments and effectiveness of interventions will be reported in the main trial results. A pragmatic approach was taken to ensure that MFFP delivery was matched to fit with usual NHS practice for each region (either primary or secondary care-led).

#### Support and audit

Throughout the pilot study, regular contact was maintained with healthcare staff to provide clinical and research support. Quality control visits were undertaken with every trained healthcare professional within a short period after training (Table 1). The independent quality control monitor observed at least one falls assessment to assess whether the healthcare professional conducted the MFFP assessment and followed the referral pathway according to protocol. A five-page quality control assessment form was completed for each visit to record issues relating to room set up, risk factor assessment, conduct of tests and onward referrals (Table 1, Intervention Fidelity). A password-protected online forum was created to allow healthcare professionals to post comments or share experiences with the research team.

## Discussion

Falls prevention strategies are complex and published research often fails to describe interventions sufficiently, making it difficult to translate findings into clinical practice. The rationale for any complex intervention should draw upon the theoretical understanding of the likely processes of how an intervention causes change, by drawing on existing evidence and theory [13]. Decision about inclusion of components within the PreFIT MFFP intervention was underpinned by evidence from high quality Cochrane systematic reviews and clinical practice guidelines. To date, based upon early findings from the PreFIT pilot study, the MFFP intervention has been found to be feasible and acceptable to participants and primary care staff.

## Conclusions

Comprehensive description of the content and testing of complex interventions is an essential component of robust trial design, delivery and reporting. We report the design and development of a complex falls prevention intervention that is currently being tested within a large-scale pragmatic trial evaluating alternative fall prevention strategies for older adults. The effectiveness and cost-effectiveness of the PreFIT MFFP intervention, compared to advice and exercise, on the prevention of falls and fractures, will be reported at the conclusion of the trial.

## Additional file

Additional file 1: The TIDieR (Template for Intervention Description and Replication) Checklist. (DOCX 28 kb)

## Abbreviations

AGS: American Geriatrics Society
BGS: British Geriatrics Society
CONSORT: Consolidated Standards for Reporting Trials
ECG: Electrocardiogram
GP: General Practitioner
HTA: Health Technology Assessment
MFFP: Multifactorial falls prevention
NHS: National Health Service
NICE: The National Institute for Health and Care Excellence
NIHR: National Institute for Health Research
PreFIT: Prevention of Falls Injury Trial
RCT: Randomised controlled trial
TIDIER: Template for Intervention Description and Replication
TUGT: Timed up and go test
